# Coping with ill-health while lacking access to health care: Acceptability of health service provision in rural Malawi – a qualitative study

**DOI:** 10.1080/16549716.2022.2062174

**Published:** 2022-05-09

**Authors:** Regina Ritter, Nego Nkhwalingwa, Carmen Anthonj, Thomas Kistemann

**Affiliations:** a University Hospital Bonn, Institute for Hygiene and Public Health, GeoHealth Centre, Bonn, Germany; b Geriatric University Hospital Bern, Bern, Switzerland; c Holy Family Mission Hospital Phalombe, Phalombe, Malawi; d Science and Earth Observation, Itc, University of TwenteFaculty of Geo-Information, Enschede, The Netherlands; e Department of Geography, University of Bonn, Bonn, Germany; f Centre for Development Research, University of Bonn, Bonn, Germany

**Keywords:** Health care system, patient-provider engagement, access and barriers to health care, Sub-Saharan Africa, SDG 3

## Abstract

**Background:**

Large parts of Malawi`s population lack access to health care. A high burden of disease, chronic poverty, and a growing population accelerate the need for extending and improving health care. One region that is struggling with service provision is Malawi´s rural district Phalombe. In addition to adequate resources, acceptability of service provision and productive patient-provider engagements are crucial determinants of health-seeking behaviour.

**Objective:**

This study aimed to better understand the interdependencies between acceptability, patient-provider engagement, and health-seeking behaviour in Phalombe. By targeting health care providers and community members, different perspectives were assessed and triangulated.

**Methods:**

Following a qualitative approach, group interviews were conducted with community members of three rural villages (n = 21) in Phalombe. Semi-structured interviews (n = 2) and a group interview among management staff (n = 3) provided insight into experiences of health care providers.

**Results:**

Community members perceived health care providers’ behaviour as disrespectful, resulting in power gaps between patients and providers. Providers blamed community members’ cultural beliefs and lack of awareness regarding health care as barriers to seek formal services. Systemic shortcomings diminished community members’ trust in service provision, while increasing frustration among providers and thus impacting patient-provider engagement. Due to insufficient resources, lack of acceptability and trust in receiving adequate services, potential patients turned into non-users of health care.

**Conclusions:**

A patient-centred approach is needed that empowers communities by involving them in health care planning, in facility management, and by raising awareness towards health issues. Trainings for providers need to focus on improving communication and building trustful patient-provider interactions. Yet, without addressing systemic constraints, providers’ frustration and patients’ lack of trust in service provision will remain and impact their health-seeking behaviour. Thus, further budget needs to be allocated to Malawi’s health care sector in order to provide resources needed.

## Background

Envisaging a world free of poverty, hunger, and disease, the United Nations (UN) formulated the 2030 Agenda for Sustainable Development. Sustainable Development Goal (SDG) 3 aims to *‘ensure healthy lives and promote well-being for all at all ages’* [1]. This includes the achievement of universal health coverage, access to essential health care services, financial risk protection, and access to safe, effective, quality and affordable essential medicines, and vaccines for all [1].

While prevalent diseases affect quality of life, productivity, and overall socioeconomic development [2], the burden of disease in a country is also directly impacted by its population’s level of access to adequate health care [3]. Access is a crucial factor for care-seeking, and to meet the demand for development in low resource settings [2].

### Health-seeking behaviour – the health access livelihood framework

Health-seeking behaviour studies provide a deeper understanding of when, why, and how people seek services, when falling ill. Obrist and colleagues [4] formulated the Health Access Livelihood Framework by applying The Sustainable Livelihood Approach [5] to health-seeking and by integrating the five dimensions of access to health care [6]. These five dimensions include the accessibility, affordability, availability, adequacy, and acceptability of services. According to the theoretical framework, the degree of access is reached along these five dimensions and depends on the interplay between health care services, broader policies, and processes on the one side, and livelihood assets that individuals can mobilise in vulnerability contexts on the other side. Thus, access increases when health care services align with patients’ needs [4].

According to this framework, knowledge and cultural beliefs about health provide perspectives on how to tackle ill-health (human capital), while social networks provide support (social capital). Land, water, and livestock (natural capital) contribute to the population’s ability to generate income (financial capital). Infrastructure, equipment, readiness of resources, and means of transport (physical capital) enable health-seeking as such. Generally, people often face hardships to access health care services when they cannot mobilise these critical livelihood resources [4].

### Malawi and its health sector

Malawi, a landlocked country with about 20 million inhabitants in Sub-Saharan Africa, has a predominantly rural population [7] and is challenged with a high burden of disease, especially of HIV/AIDS and malaria [8]. The chronic poverty among large shares of the constantly growing population accelerate the need for improved health care (Table 1). One of the areas that is struggling with the provision of health care is the rural district Phalombe in Malawi’s south. When falling ill and deciding to seek health care services, people in rural Malawi face several hardships in terms of access, that were introduced as the dimensions of access [6] within the Health Access Livelihood Framework [4]. These include particularly spatial inaccessibility [9], financial vulnerability of the population at risk [9,10], and lacking resources within the facilities [8]. In addition to these systemic shortcomings, acceptability of service provision determines access and health-seeking behaviour among population groups. Acceptability comprises the fit between lay and professional health beliefs, provider-patient engagement, and the extent in which health care provision determines patients’ responses to services [6,11]. Especially in settings with limited access and low level of health literacy, trust in health providers is crucial [11].

**Table 1.** Population and health indicators of Malawi [7,14–16]

	Population and health indicators	Malawi	Southern Region	Phalombe
**Population**	Total population (in 1000) [2018]	17,564	7,751	429
	– Population < 15 years	43.9%	44.5%	/
	– Population > 65 years	3.7%	3.7%	/
	Population growth rate [2018]	2.9%	2.8%	2.9%
	People in rural areas living below national poverty line [2017]	59.5%	65.2%	83.2%
	unemployment rate (age 15–64 years) [2018]	18.5	16.7	14.6
	illiteracy rate (aged 5 and older) [%] [2018]	31.4	33.0	35.0
**Health Indicators**	Density of medical doctors (per 10,000 population) [2010–2018]	0.4	/	/
	Life expectancy (years) [2019]	64.2	/	/
	Total fertility rate (children/ woman) [2015–16]	4.4	4.6	5.0/
	Maternal mortality rate (per 1,000 live births) [2017]	0.349	/	/
	Under-5-mortality rate (per 1,000 live births) [2015–16]	50.0	/	/
	HIV prevalence (age 15–49 years) [2015]	8.8%	12.8%	15.5%

Acceptability, patient-provider engagement, and health-seeking behaviour *per se* have been investigated in previous studies in different settings [10–13]. A research remains on acceptability in Phalombe district. Our study intends to fill this knowledge gap, and to contribute to a better understanding of the interdependencies regarding acceptability, patient-provider engagement, systemic shortcomings, and health-seeking behaviour in a rural setting in Malawi by reporting perspectives of health personnel and community members, who are rarely given the chance to contribute towards health policy [9].

The guiding research questions of our study were:

- What determines acceptability of service provision and patient-provider engagement?
- How do acceptability and patient-provider engagement determine health-seeking behaviour in Phalombe district?
- Which interdependencies can be found between acceptability of service provision and systemic constraints?
- What implications do the results have for the achievement of SDG 3 in areas with limited access to health care services?

## Methods

### Study site

Malawi is divided into three administrative regions – the Northern, Central, and Southern Region – and 28 districts, with substantial urban-rural and regional differences regarding socioeconomic factors. The Southern Region has the highest population density, poverty in rural areas, illiteracy, and HIV prevalence [7,16].

**Figure 1.**
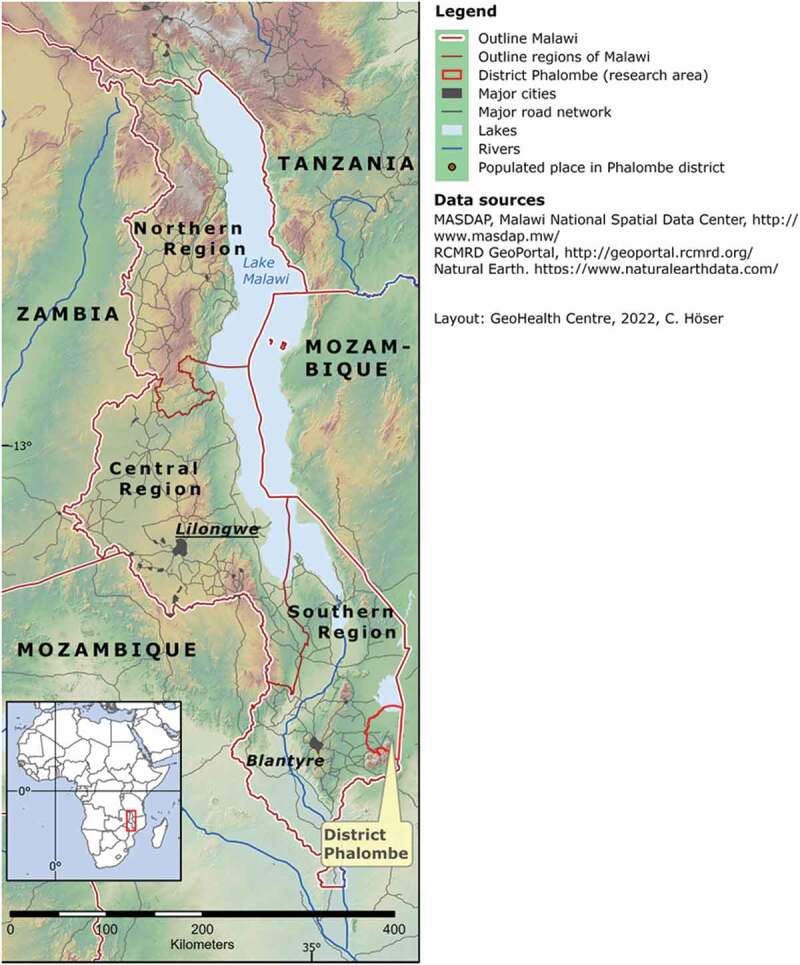
Study area Phalombe district in Southern Region, Malawi [17–19].

Our study was carried out in Phalombe district (Figure 1) which serves as a model case for the Southern Region or other rural areas across Malawi.

Generally, maternity facilities, health centers, and rural hospitals, provide health care of the primary level, such as curative and preventive services [8]. The main health center is located in Phalombe City and owns enhanced equipment compared to Phalombe´s other health centers. However, it lacks an operating theatre or diagnostic equipment such as an x-ray apparatus or sonographic units. One non-profit private hospital, the Holy Family Mission Hospital (HFMH), provides secondary health care services. It serves the more complicated cases of the entire district including Caesarean sections or abdominal surgeries.

### Data collection tools

This study followed a qualitative approach, with data collected through semi-structured and group interviews from October to November 2016 in Phalombe. Three group interviews were conducted with community members (n = 21) of the villages Nalingula, Njumwa, and Mariko. Group interviews facilitate the investigation of different viewpoints of groups and individuals within one cost-effective interview [20,21]. Predefined questions initiated the interviews regarding difficulties in accessing health care in general, perceived acceptability of service provision, and patient-provider engagement.

Semi-structured problem-oriented interviews with health workers (n = 2) and a group interview among the management of the HFMH (n = 3) gave insight into experiences of health care providers. The interviews followed an interview guide based on key questions to create a structure for data collection and analysis [21]. The key questions focussed on access to health care including acceptability of service provision and potential improvements in accessing services. The method left enough room to ask additional questions and include further topics, helping to capture in-depth information, experiences, and subjective perceptions [22].

The group interview with the HFMH management staff was conducted after presenting preliminary results to them. This debriefing aimed to discuss results and questions coming up during research, while gaining further input for a critical reflection on the study’s outcomes from administrative units of the hospital. Again, key questions focussed on access to and acceptability of health care services.

### Participants

The target population for group interviews within the villages belonged to the poor rural population, depending on subsistence farming for their livelihood. The community members lived outside the catchment area of the private HFMH (8 to 11 kilometres), but had a governmental health center closer by (2 to 4.5 kilometres). Thus, services within health centers became the focus throughout all interviews. Community members were sampled through the village headmen, who randomly asked six to eight male and female patients from different age groups and households to participate (Table 2). Following the assumption that community members are rarely given the chance to contribute towards health policy debates in their country, the explicit focus of our study was to capture, analyse and report their perspective.

**Table 2.** Summary of data collection

Data collection method	Qualitative data collection		
	Group interviews		Semi-structured interviews
Study population	Patients in 3 villages (Nalingula, Njumwa, Mariko) in rural Phalombe; n = 21 (6 male, 15 female) aged 16–61 years	Management Board of HFMH n = 3 (2 female, 1 male) aged 38–52 years	Health personnel n = 2 (1 male clinical officer from HFMH, 1 female nurse from main health center) aged 33–59 years
Sampling by	Village headman	Researcher	Researcher
Collected data	Perspectives of community members regarding acceptability, patient-provider engagement, resulting health-seeking behaviour	Predefined key questions to address health providers´ point of view In-depth information, experiences, and regional knowledge	

Barriers regarding affordability and spatial accessibility were supposed to play a minor role within the interviews as the villages were located within a five-kilometres-range to a health care facility that offered services free of charge. Participants of the group interviews within the communities will be presented as ‘patients’ in the following passages.

Semi-structured interviews were conducted with a male clinical officer from the HFMH and a female nurse from Phalombe´s main health center. Sampling of the participants occurred through the first author after getting to know the facilities and its providers. Sampling based on the providers´ willingness to share information. By selecting interviewees from different facilities, sex, age groups, and cadres of personnel, a broader picture was supposed to be painted (Table 2).

The group interview with the management board included three participants, aged between 38 and 52 years. These groups are presented as ‘providers’ in the following.

### Data collection process

The field research was implemented by a team of two, one of whom was fluent in English and Chichewa, the national language in Malawi. Being a student of the College of Nursing in Phalombe, he was part of the health care system and familiar with the facilities and the district.

The group interviews with the patients were held in Chichewa as not all patients spoke English. Detailed notes were taken simultaneously while key inputs were summarised at the end of every interview. They lasted 35 minutes in average and were conducted in a private environment, safeguarding confidentiality and anonymity.

Data collection among providers occurred in English. The interviews took 27 and 19 minutes, while the group interview with the hospital’s management took 64 minutes.

Qualitative data was audio-recorded and transcribed directly after conduction. Transcripts were compared with the original audio record and discussed within the research team.

### Data analysis

In order to analyse the empirical qualitative data, open coding was applied. Based on the main issues addressed by the study participants, empirical data was accessed multiple times to identify themes. Axial coding was used to define categories and draw connections between the codes and sub-codes (Table 4).

**Table 3.** Characteristics of study population

	Group interviews among community members	Group interview among management	Semi-structured interviews personnel
Male participants	n = 6 (28.6%)	n = 1 (33.3%)	n = 1 (50%)
Female participants	n = 15 (71.4%)	n = 2 (66.6%)	n = 1 (50%)
Age range (years)	16–61	38–52	33–59
Mean age (years)	35.5	45	46

**Table 4.** Categories, codes, and sub-codes (for more detailed information, see Supplementary Material Table S1)

Category	Characteristics of providers				Characteristics of patients			Systemic constraints				
Code	Behaviour		Communication		Awareness		Empowerment	Availability			Accommodation	
Sub-code	Waiting time	Favouritism	Violent language	Disrespect	Cultural context and Health beliefs	Recognition of illness	Empowerment	Equipment/ diagnostics	Drugs	Personnel/ workload	Basic amenities	Capacities for admission

Qualitative data from patients and providers were triangulated against one another and against literature to validate empirical findings [23]. Anonymity and confidentiality on the transcripts were secured by using subject identifier codes. Quotes of patients used codes consisting of the village, sex (m/f), and age of the patient. Quotes of health personnel and the hospital’s management will be presented as ‘provider’.

## Results

Patients were between 16 and 61 years of age, while the majority was female. Table 3 shows further characteristics of the study population regarding sex and age.

Based on the main issues addressed by the study participants, Table 4 displays categories, codes, and sub-codes that were defined based on the empirical data.

### Characteristics of providers – behaviour and communication

Patients addressed long waiting times and perceived providers to ‘*just play with their phones, laptops, and friends without attending patients in time*.’ (Mariko, f 40). Due to long waiting times, patients reported serious aggravations of medical conditions while lining up for consultation.

Providers described long waiting times and bad attitudes towards patients as a consequence of understaffing and high workloads, especially when one provider had to carry out various responsibilities. Besides, the management staff acknowledged that limited communication increased patients’ perception of being left alone and treated disrespectfully. In addition, patients criticised the attitude and behaviour of providers as often prioritizing friends, relatives, and wealthy people when attending patients. As a consequence, patients *‘stayed at home although [being] in severe pain’* (Mariko, f 40). One provider validated this perceived favouritism and defined it as a *‘privilege of working in a hospital’* to treat relatives first. Furthermore, he acknowledged that many health workers did not have empathy with the poor.

Within all group interviews, patients criticised providers’ disrespectful language and behaviour, when yelling at patients and not listening to their needs. It was reported that ‘*nurses shouted at [pregnant] women and sent them out of the delivery room*’ (Nalingula, f 59).

### Characteristics of patients – awareness and empowerment

All providers criticised patients’ *‘ignorance of their own health’*, when waiting too long to seek health care or when seeking services from a traditional healer rather than a formal health facility. Providers addressed beliefs within communities that prevented the provision of services in a timely manner, causing complications of treatment. One belief surrounding delivering mothers, for instance, was to wait until darkness to seek birth assistance rather than walking outside during daylight once labour had started. *‘So, when labour starts during the day, women stay at home and wait until night’*. Thus, a provider called for ‘*massive civil education’.*

Patients described issues arising from the lack of empowerment as *‘there [was] nowhere to go’* (Mariko, m 49). So far, attempts to address their problems to providers did not improve their situation. According to a provider, further issues regarding empowerment concerned women within the communities as they were obliged to ask authorisation from a male family member before seeking health care services. *‘So [women] end up at the hospital when things get really complicated’.*

### Systemic constraints – availability and accommodation

Systemic constraints were addressed within all interviews. For instance, inadequate equipment and services hampered sufficient diagnostic and treatment of patients, while leading to frustration among health care providers. As reported by providers, a lack of equipment forced them to improvise and treat patients based on assumptions, which decreased patients’ safety and quality of care. A lack of medication in health care facilities forced patients to buy their medication out-of-pocket from the informal private sector or to go home without treatment. According to providers, as a consequence of inadequate resources and manpower, patients failed to seek care at a facility as they expected not to receive adequate services.

The absence of basic amenities and low capacities for admission, including opening times of facilities and the number of beds for accommodation, posed further systemic constraints that were addressed by patients and providers. Missing beds caused *‘patients to sleep on the floor’*, while ambulances were *‘unable to collect patients, because there [was] no fuel’* [provider]. Lacking electricity and blackouts led to disruptions in providing diagnostic services or even emergency surgeries. Thus, patients could not receive the services they needed.

## Discussion

Our empirical study suggests that acceptability of service provision and patient-provider engagement are shaped by perspectives, characteristics of personnel and patients, previous experiences of interaction, and systemic constraints. Patients targeted by this research perceived health care providers to exercise power through their communication practices, misbehaviour, and language used to explain health problems. Poor patient-provider interactions adversely affect health-seeking behaviour and lead potential users to give up on the health care system, as shown here and in previous studies [9,11,12,24]. The patients’ reliance on providers and their inability to make their own voices heard in medical care settings increased their feeling of helplessness and fear to seek services [13].

Providers interviewed in our study criticised patients’ cultural beliefs and lack of awareness to seek formal health care in time. Thus, close communication between providers and community members needs to overcome information barriers and create awareness regarding service provision. In order to enable community members to recognise their own health status and to take appropriate action, human capital including local knowledge and education need to be mobilised [4].

Empirical data showed providers’ lacking empathy with the poor and their favouritism in treating wealthy people or family members. As indicated in our research and literature, gender inequality was another barrier for women to access services, as married women were less likely to secure funds for health care from family members compared to men [25]. Generally, acceptability and trust barriers seem to be disproportionately faced by socially disadvantaged groups, which has an impact on the justice of health care distribution and demonstrates social inequality within health care [11]. To cease blaming patients for poor health-seeking behaviour, providers need to be educated about the beliefs and realities of communities. Trainings tailored to providers need to improve their communication skills, emphasising the importance of a trustful patient-provider interaction that takes into account the patients’ circumstances seriously. A previous Malawi-based study indicates that encouragement to ask questions during consultations, privacy, and confidentiality issues enhance patients’ perceived quality of care [26]. Especially in a setting with a low level of awareness and access to health care, trust in health workers is largely influenced by respect, assurance of treatment, and explanations on examinations and procedures [11,27,28]. By addressing interactions between patients and providers, vulnerabilities of the population-at-risk regarding social capital can be tackled [4].

Generally, a more patient-centred approach is needed to empower individuals and communities and make their voices be heard, as participation can help to reduce power gaps between the health providers and the population they serve [29]. Socially excluded people need to be reached by involving them in designing, implementing, and assessing interventions to ensure that they are socially acceptable and accessible to everyone [30,31]. Systems for supervision, feedback, and accountability of health providers might empower patients and increase acceptability for health care services [4,28]. As acknowledged by Malawi’s Ministry of Health, communities were more likely to contribute to the improvement of service delivery, when they felt involved in the facility’s management [8].

Moreover, interdependencies between acceptability of services provision and systemic constraints regarding availability of resources become apparent within this study.

Obtaining financial and physical capital in the form of means of transport, numbers of facilities, and readiness of sufficient resources represent further preconditions to access quality health care [4,32]. Systemic constraints have a major impact on patients’ perceived appropriateness, therapeutic relationships, and trust in service provision as suggested by our research and previous studies [4,33]. Within health care facilities in Phalombe district, readiness of equipment, drugs, and appropriate services were perceived as insufficient. Low capacities for hospital admission increase these constraints. Thus, lacking availability of resources diminished acceptability and trust in service provision, while reinforcing patients to stay at home when feeling sick, as they expected not to receive adequate treatment in health care facilities.

However, acceptability does not only imply the responsiveness of health care provision to patients’ needs, but as well the ways in which features of the system influence providers’ behaviour towards patients [11]. Insufficient diagnostic equipment, inadequate basic amenities, and manpower impact the working environment of health personnel in Phalombe, leading to disruptions in service provision, frustration, and unethical behaviour. Our empirical findings are in line with previous research, describing poor relationships with other staff, inadequate equipment to treat patients sufficiently, low salaries, lacking response from hospital administration, and low staff numbers to create a high burden of stress and poor working conditions [13,33–35]. Though providers may not have control over systemic constraints, they carry some responsibility of the patient’s clinic experience. Each provider is capable of controlling his or her own attitude and treatment of patients [13]. More insight is needed regarding factors that facilitate compassionate health care provision in resource-poor settings. Yet, without addressing the availability of resources needed, difficulties to change the health providers’ behaviour towards patients will remain as health providers are often just the representatives of the system in which they work [11]. The allocation of further budget for the health care sector is required to provide the resources and training required [9].

Yet, some vulnerabilities remain beyond the scope of action of the health care sector.

To advance the current model of health service delivery, which, as has been shown for other Sub-Saharan countries, is argued to be still built on the colonial model [36], arrangements that strive for strengthening of community participation are essential. The current scarcity of resources can be seen as a result of a long history of national disinvestment and uneven health care policies [33]. To improve outcomes for the most vulnerable, equity as a core principle is required to antagonize neoliberal associations with wealth status of care-seekers [37]. According to the WHO ‘Health in all Policies’ strategy [38] it calls for the development of integrated solutions. Broad approaches to primary health care, education, legal and social protection, women’s empowerment, water and sanitation, transport, and communication are essential [30,32].

### Strengths and limitations of this study

This study gives rise to limitations with respect to several issues. The sample sizes of patients and providers were relatively small, with participants representing just few out of many ethnic groups that may or may not seek public health services. The sampling strategy by village headmen may be subject to social desirability bias. This study did not include education, and socioeconomic status of the patients, as well as utilisation of traditional medicine or informal services. These topics may have allowed for deeper and more detailed insights. The selection of providers was based on personal impressions concerning their willingness to share information. Thus, the number and subjective choice of the participants might have influenced the outcomes. As views and opinions are related to the discussion’s context, findings are not generalisable *per se* for people in Phalombe or whole Malawi [39]. However, empirical data has been triangulated with literature, showing similar trends to the ones we revealed. As findings were generally consistent within the interviews and literature, they appear to be representative for the Southern Region and characteristic for a larger number of rural settings in Malawi.

Further potential bias was rooted in the group interviews among community members, which were held in Chichewa and translated into English. These translations might have caused a loss of context-specific expressions. By extensive briefing and training prior to the data collection, this potential limitation was reduced as much as possible. Generally, different cultural backgrounds might have caused a loss of information or misinterpretation concerning cultural aspects and context-specific contents. Subjective interpretations and unconscious assumptions, arising from the cultural background and norms of the researchers, might have influenced the processes of data collection, analysis, and evaluation.

Yet, by including various stakeholders and triangulating empirical findings, different perspectives and views were gathered. Despite the relatively small sample sizes and inconsistent sampling procedures, a wide-ranging overview was gained. This provided a better understanding of the realities among Phalombe’s community members and health professionals.

Generally, an in-depth investigation including all dimensions of access could have provided a broader overview regarding the hardships in accessing health care services in Phalombe. However, the explicit focus of this research lied on acceptability of formal health care services and its impact on health-seeking behaviour. Interdependencies between the dimension acceptability and further systemic hardships have been indicated without claim to be complete.

## Conclusions

This study aimed to contribute to a better understanding of acceptability regarding formal health care services, patient-provider engagement, and resulting health-seeking behaviour in rural Malawi. The findings suggest that poor patient-provider interactions and power gaps between the health providers and the population served weaken the acceptability of health care services. Furthermore, interdependencies between acceptability and availability of resources become apparent as systemic constraints impact trust in qualitative service provision, working conditions for health personnel, and therefore patient-provider engagement. The resulting distrust in service provision affected patients’ health-seeking behaviour and led them to give up on the health care system.

To achieve SDG 3 and ‘*ensure healthy lives and promote well-being for all at all ages*’ [1], a patient-centred approach for health care service provision is needed that empowers individuals and communities. By addressing health literacy and by involving communities in health care planning, power gaps between patients and providers can be reduced while acceptability for health care services can be raised, and health services overall can be improved. In order to address productive communication that is responsive to patients’ needs, the management of health care facilities could offer specific trainings for providers that tackle their communication skills and emphasise the importance of a trustful patient-provider interaction.

Yet, without addressing systemic constraints, providers’ frustration regarding their working environments and patients’ lack of trust in service provision will remain. Tackling availability and acceptability in rural Malawi requires further budget for the health care sector in order to provide the resources needed.

## Supplementary Material

Supplemental Material

## Paper context

Few studies have been conducted to evaluate the level of acceptability towards health service provision in rural Malawi. Our research intends to contribute to a better understanding of acceptability, patient-provider engagement, and their effects on health-seeking behaviour by presenting perspectives of community members and health care providers in the Phalombe district. Findings suggest the need for a patient-centred approach, while addressing systemic constraints to enhance providers´ working environment and patients’ trust in service provision.
