# Supplementary material for: Coping with ill-health while lacking access to health care: Acceptability of health service provision in rural Malawi – a qualitative study
Source: Glob Health Action. 2022 May 9;15(1):2062174. doi: 10.1080/16549716.2022.2062174 (PMC9090431; doi:10.1080/16549716.2022.2062174)
Supplement: Supplemental Material [file ZGHA_A_2062174_SM0206.docx]

**Supplementary material:**

Table 1 Results of empirical research coded in categories, codes, and sub-codes

| **Category** | **Code** | **Subcode** | **Empirical findings** | | |
| --- | --- | --- | --- | --- | --- |
|  |  |  | **Patients** | **Personnel** | **Management** |
|  | **Behaviour** | Waiting time | “Sometimes patients even die in the queue because of not being attended in time.” (Nalingula, f 20)  “We wait a very long time without being attended.” (Mariko, f 31)  “They [staff] just play with their phones, laptops and friends without attending patients in time.” (Mariko, f 40) |  | “There are two clinicians and one of them being the anaesthetist. When there is a procedure in the theatre, he is supposed to leave the patient and attend the procedure. What happens to the patient? He sits there and waits.”  “Concerning the long waiting hours in the radiology. We only have one radiographer. So when he is off duty, the patient is sent back or has to wait” |
| Characteristics of health personnel |  | Favouritism | “Some doctors or nurses don’t attend us but their friends and relatives first.” (Nalingula, f 59)  “Most of the times, health workers attend people that they know first. Especially when they are rich. (Njumwa, f 46)  “Sometimes, due to the attending of health workers, we just stay at home although we are in severe pain.” (Mariko, f 40) | “In each and every institution, if you are a relative of the health personnel and you are sick, then you don’t line up the way other patients do. That is their privilege of working in the hospital.”  “Lots of the health workers do not have empathy with poor people.” |  |
|  | **Communication** | Violent language | “Concerning pregnant women, the health workers shout at them and leave them alone. Sometimes women deliver babies without supervision of the health worker. (Njumwa, f 35)  “Nurses shout at women and send them out of the delivery room. The women sometimes deliver babies outside the ward… [There is a] lack of kindness and respect to patient.” (Nalingula, f 59) |  | “Maybe here in Holy Family, shouting at patients is not an issue. But in governmental facilities, it is an issue. A lot of the patients run away from the governmental facilities and come here, because they are treated well.” |
|  |  | Disrespect | “Some of the health workers […] yell at patients and throw their health passport back at patient while the he is still explaining his condition to him. (Njumwa, f 32) |  | “Sometimes we don’t explain the patient what is happening. We just leave the room.” |

| **Category** | **Code** | **Subcode** | **Empirical findings** | | |
| --- | --- | --- | --- | --- | --- |
|  |  |  | **Patients** | **Personnel** | **Management** |
| Characteristics of patients | **Awareness** | Recognition of illness |  | “These people come from very far away to the health centers. And most people don’t come straight to the hospital, when they are sick. […] Others buy drugs at the drug store for treating fever or whatsoever before they go to the hospitals. Others have fears to go to the hospital at all.” | “[Patients] stay and wait at home until they are very very sick. But if the community had the habit to come to the hospital before the condition has worsened, maybe some of these issues wouldn’t get such a big problem.” |
|  |  | Cultural context & health Beliefs |  | “There are beliefs in the villages, that […] they should go to traditional healers. That this illness should not be treated in a hospital, [but] traditionally. I think this is ignorance of their own health.”  “I think there should be massive civil education to tell the people that the only place to go, when they are sick, is a hospital, not the traditional doctors.” | “When we are sick, the first priority for us is to go to an African, a traditional doctor. When the situation worsens afterwards, that´s when they go to the hospitals. This belief, this habit needs to be abolished. Another belief is, when I am pregnant and labour starts, I should not walk during the day to go to the health facility. […] So, when labour starts during the day, women stay at home and wait until night.” |
|  | **Empowerment** | Empowerment | “There is nowhere to go. We have been meeting about this problem on Monday when the health centre opened. But they [staff] just shout at us” (Nalingula, f 56)  “Sometimes we go to health officers [to report our problems] but there is no change” (Mariko, m 49) |  | “There are issues that my uncle or whoever has to authorize me to go to the hospital […] If my baby is sick, I cannot just decide on my own to go to the hospital […] There are small issues, small beliefs, that prevent us from receiving antenatal care in time. So, we end up at the hospital when things get really complicated.” |

| **Category** | **Code** | **Subcode** | **Empirical findings** | | |
| --- | --- | --- | --- | --- | --- |
|  |  |  | **Patients** | **Personnel** | **Management** |
| Systemic constraints | **Availability** | Equipment/ diagnostics | “[Phalombe health center] is facing many problems like inadequate equipment and lack of room for patients“(Nalingula, f 59)  “There is no adequate equipment, a lack of full investigation to find out, what the patient is suffering of. Sufficient treatment is only given to rich people. We face a problem about deaths of patients due to inadequate treatment or care at health centres. (Njumwa, f 22) | “For us to work effectively, we need all the necessary resources to be available. Sometimes we just don’t have the resources, we only improvise, which is not good for our safety, for the patients´ safety, for the quality of care. We have limited investigative mechanisms. So, we […] just treat based on assumptions, based on signs and symptoms. You need a good working-environment, you cannot just work all frustrated.” | “We only attend emergencies right now, no elective cases. Why? Resources!” |
|  |  | Drugs | “We are told that the drugs which are prescribed are not available in that particular health centre. So, they direct us to the private sector to buy them there. This is a big problem for us (Nalingula, f 24) | “The other thing is a lack of adequate health services in terms of resources and even manpower. When they go the health facilities and the health workers tell them “you´re suffering from this and that and the proper drug for this disease is not available”, these patients just go back home. Or they find that the clinicians or the nurses are not around because they have been going to a workshop.”  “Even the issue of inadequate health-personnel is a national-wide thing. The government doesn´t have enough money to put the people on payroll.” | “Sometimes the patients even know that there is no funding. So, they just sit at home. They know, if they go to the hospital, they will be told that there is no drug, no this no that. So, the people just sit at home without going to the hospital.” |
|  |  | Personnel/ workload |  |  | “I think the government needs to put more staff into the health system […] One of the contributing factors [to bad attitudes] is the workload.”  “You find a lot of students working here. The quality that is expected is not achieved because they are still students. Qualified staff is really an issue.” |
|  | **Accommodation** | Basic amenities |  | “We need enough fuel. Sometimes the ambulance is unable to collect the patients, because there is no fuel.”  “We don´t have a generator, only a solar lamp. The whole maternity ward doesn’t have electricity due to bad construction. It is difficult to do sutures or to deliver a baby without any light. ” | “Those frequent blackouts... Can you wait for the lab or the lights to come? The patient that needs a procedure is told “Wait, maybe the power will come back.” Or “Come next week”. Sometimes a patient stays here for one week without being operated due to blackout.” |
|  |  | Capacities for admission | “Guardians sometimes have to sleep outside the ward on the floor” (Nalingula, f 24)  “During lunch time, they close the [facilities] early while we still wait in the queue” (Njumwa, f 35)  “They open later than they should.” (Njumwa, 3 participants) | “We have a problem with beds, too. Beds and sheets are not enough for the patients.” | “Patients are sleeping on the floor. That cannot be a healthy situation.” |
